# Activity of Clinically Relevant Antimalarial Drugs on *Plasmodium falciparum* Mature Gametocytes in an ATP Bioluminescence “Transmission Blocking” Assay

**DOI:** 10.1371/journal.pone.0035019

**Published:** 2012-04-13

**Authors:** Joël Lelièvre, Maria Jesus Almela, Sonia Lozano, Celia Miguel, Virginia Franco, Didier Leroy, Esperanza Herreros

**Affiliations:** 1 GlaxoSmithKline R&D, Tres Cantos Medicine Development Campus, Malaria Discovery Performance Unit, Madrid, Spain; 2 Medicines for Malaria Venture, Geneva, Switzerland; University of Melbourne, Australia

## Abstract

**Background:**

Current anti-malarial drugs have been selected on the basis of their activity against the symptom-causing asexual blood stage of the parasite. Which of these drugs also target gametocytes, in the sexual stage responsible for disease transmission, remains unknown. Blocking transmission is one of the main strategies in the eradication agenda and requires the identification of new molecules that are active against gametocytes. However, to date, the main limitation for measuring the effect of molecules against mature gametocytes on a large scale is the lack of a standardized and reliable method. Here we provide an efficient method to produce and purify mature gametocytes *in vitro*. Based on this new procedure, we developed a robust, affordable, and sensitive ATP bioluminescence-based assay. We then assessed the activity of 17 gold-standard anti-malarial drugs on *Plasmodium* late stage gametocytes.

**Methods and Findings:**

Difficulties in producing large amounts of gametocytes have limited progress in the development of malaria transmission blocking assays. We improved the method established by Ifediba and Vanderberg to obtain viable, mature gametocytes *en masse*, whatever the strain used. We designed an assay to determine the activity of antimalarial drugs based on the intracellular ATP content of purified stage IV–V gametocytes after 48 h of drug exposure in 96/384-well microplates. Measurements of drug activity on asexual stages and cytotoxicity on HepG2 cells were also obtained to estimate the specificity of the active drugs.

**Conclusions:**

The work described here represents another significant step towards determination of the activity of new molecules on mature gametocytes of any strain with an automated assay suitable for medium/high-throughput screening. Considering that the biology of the forms involved in the sexual and asexual stages is very different, a screen of our 2 million-compound library may allow us to discover novel anti-malarial drugs to target gametocyte-specific metabolic pathways.

## Introduction

Recent years have witnessed renewed impetus for malaria control but this disease is still leading to nearly 1 million deaths annually. This is why the Malaria Eradication Research Agenda (malERA, http://malera.tropika.net) initiative, created in 2007, re-established the long-term goal of malaria eradication. Spread and maintenance of the malaria parasites rely on their transmission between humans and *Anopheles*. Attempts at eradication should therefore include transmission-blocking approaches aiming at identification of therapies capable of eliminating *Plasmodium* gametocytes, the sexual forms of the parasite. To test potential anti-gametocyte drugs, both an improved gametocyte production method and a reliable assay to assess activity of compounds against mature gametocytes are required.

The biology of gametocytes is complex and consists of five morphologically identifiable stages: stages I to IV (immature gametocytes) sequestered into human tissues; and stage V, the infectious sexual form that circulates in the blood-stream. Despite some attempts to gain insights into the biology of the parasite's gametocytes and to identify potential drug targets within their proteomes [1], identification of new molecules blocking the production of these forms relied mainly on phenotypic assays and recently on a small number of novel assays using ATP detection [2], flow cytometry [3], stage-specific markers such as Pfs16 [4], [5] or both of the latter two methods [6].

Moreover, the renewed interest in parasite transmission has highlighted a crucial need for additional tools. Difficulties in producing large amounts of this form have limited research progress in the development of blocking transmission assays. Using the method of Trager and Jensen, Smalley was the first to attempt to obtain sexual forms of the parasite, but these gametocytes were not, or were rarely and unpredictably functional, even when they were allowed to develop for a longer time [7]. In 1981, Ifediba and Vanderberg improved the method by adding hypoxanthine to the culture medium and obtained mature *P. falciparum* gametocytes [8]. It later became possible to remove the asexual forms in most gametocyte production protocols by introducing N-acetyl-D-glucosamine (GLcNA), a sugar that completely blocks invasion of the erythrocyte by this parasite but does not affect gametocyte maturation [9]. More recently, Mann found that addition of bistratene A, a protein kinase C inhibitor, also inhibited merozoite invasion and reportedly induced gametocytogenesis [10]. Moreover, Fivelman et al [11] established a useful but complex protocol to obtain gametocytes reaching different maturation stages. All of these methods have a low production rate and suffer from costs and reproducibility problems because culture medium is currently supplemented with human serum in different proportions.

Currently, we lack effective activity assays against late stage gametocytes. The gold standard test relies on dose-response measurement by manual microscopic counting of the number of gametocytes after drug exposure at different concentrations. This method is time-consuming and subject to human error. Flow cytometry has been proposed to assess the *in vitro* gametocytocidal activities of potential anti-plasmodial drugs [3] but each well has to be processed on costly equipment. Whilst an improvement on the light microscopy method, the use of cytometry does not allow for large-scale assessment of compound activity on late-stage gametocytes. Another promising assay, which has recently been developed [2], is based on detection of fluorescence of reduced alamar blue. Nevertheless, several important drawbacks to its routine use have been reported [12] such as the amount of gametocytes required, and the targeted stage which is not confined to mature gametocytes. The ATP bioluminescent assay remains a gold standard to explore parasite viability. ATP plays a central role in energy exchanges in biological systems (both eukaryotic and prokaryotic), serves as the main donor of free energy, and is produced in all metabolically active cells. This parameter is currently used as a tool to assess the functional integrity of living cells. Injury and death result in a rapid decrease in cytoplasmic ATP, [13] which therefore provides a reliable platform to test the effect of small molecules on the ability of gametocytes to develop. An assay based on ATP has in fact been published recently. However, methodological flaws can be noted; a crucial one is the use of frozen gametocytes [14].

We report an innovative solution to produce and purify mature (stage IV–V) gametocytes and to develop and validate a method based on bioluminescence-mediated detection of ATP as the preferred measure of gametocyte viability. This assay has been validated successfully and for the first time provides extensive activity data of 17 clinically relevant antimalarial drugs.

## Results

### Gametocyte production protocol

Capitalizing on previous studies, we developed a method which overcomes some significant constraints. We started to use lipid-rich bovine serum albumin concentrate (AlbuMAX II) instead of the standard supplement, human serum. AlbuMAX is much cheaper and avoids variability between the different serum batches and donors, thus increasing the reliability of the assay. We also kept adding hypoxanthine to the basal medium throughout the process and added *N*-acetyl-D-glucosamine and bistratene A during the last phase of gametocyte differentiation to obtain approximately 1–2% gametocytemia (stage IV and V) between day 15 and day 18.

**Figure 1:**
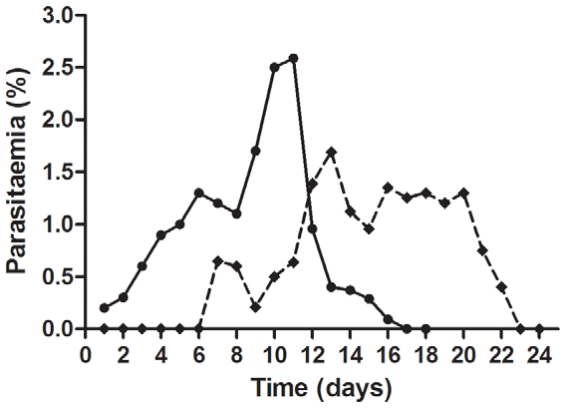
Kinetics of parasitaemia (asexual stages only, black dots) and gametocytaemia (stages 1–5; black squares) by microscopic observation, represented as a percentage of total erythrocytes. The number of asexual forms increased up to maximum parasitaemia on day 11. From that time onwards, a rapid decrease occurred leading to a parasitaemia (asexual stages) close to zero on day 17. Sexual forms were first detected on day 7, and gametocytaemia reached a peak of 1.69% on day 13. The gametocytes, initially in early forms, developed into mature stages reaching stages IV and V from day 15 to 20. After day 15, more than 70% of the gametocytes are in stage IV–V.

Under these conditions, different strains of *P. falciparum* (3D7A, W2, NF54, Dd2 and 3D7HT-GFP) were tested to determine which one was the most appropriate. Differences in gametocyte production were seen among these strains. The *P. falciparum* 3D7HT-GFP strain [15], that constitutively expresses green fluorescent protein (GFP), showed the highest sexual differentiation ratio and the greatest ability to mature and was therefore used to set up the assay. The gametocytogenesis profile of the 3D7HT-GFP strain is shown in Figure 1.

### Purification process

**Figure 2:**
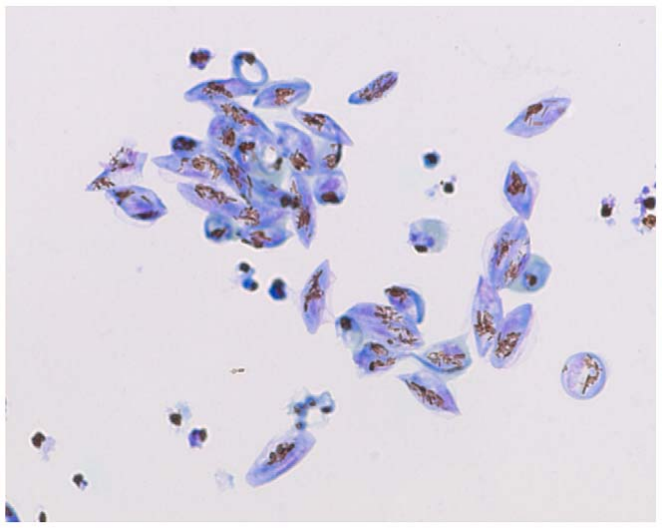
Giemsa-stained culture of gametocytes on day 15 after both Nycoprep and magnetic purification steps, (1000×).

Our gametocyte culture is composed of 1–2% mature gametocytes but also 98–99% red blood cells (RBCs), which contain a significant intracellular ATP concentration that interferes with the readout. It was therefore necessary for RBCs to be removed from the culture. Stage IV–V gametocyte cultures were enriched on a Nycoprep cushion, based on density differences between gametocytes and RBCs. Most RBCs were depleted, but an accurate quality control of samples showed that an unacceptable number remained. An additional purification step, consisting of magnetic column separation, was thus performed. The ability of all *Plasmodium* erythrocytic species to degrade hemoglobin (a Fe(II) diamagnetic complex) to hemozoin (a Fe(III) paramagnetic complex) [16] was used, making discrimination between retained gametocytes and eluted RBC possible when the magnetic field was applied. We thus managed to obtain a culture containing more than 70% stage IV–V gametocytes (Fig. 2). We were then concerned by the viability of gametocytes after this two-step purification. Intracellular ATP level, morphology, internal motility and response to exflagellation stimuli were therefore all checked (data in annex) at T = 0 h and T = 48 h, and the results showed that our purification approach did not significantly affect either viability or exflagellation of gametocytes throughout the purification procedure.

### Assay development and validation

Following successful mass production of viable gametocytes, an assay for large-scale testing of the activity of compounds against stage IV–V gametocytes, by measuring ATP levels after drug exposure, was developed. Gametocytes ATP intracellular levels were measured by the luciferin-luciferase methodology.

**Figure 3:**
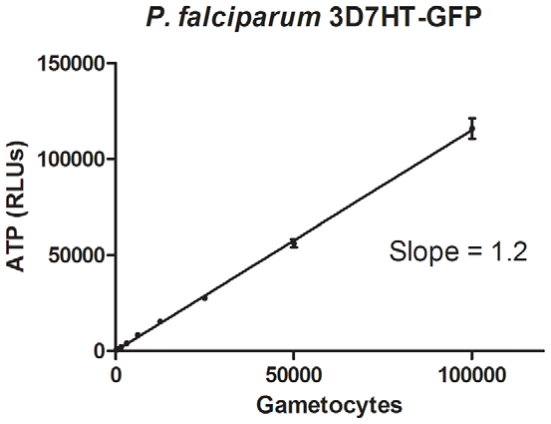
ATP level per gametocyte number (R^2^ = 0.99) counted with the luminometer after serial dilution. Gametocytes were counted using a Neubauer chamber. Each point represents mean values of 3 replicates ± SD.

Firstly, to assess the robustness and the quality of the ATP bioluminescence assay we calculated the Z′-factor, the screening window coefficient (calculated with the control data) [17]. Values between 0.5 and 1.0 indicate excellent assay quality. The Z′-factor for the assay (± standard deviation) was 0.68 (±0.02). To determine whether ATP quantification provided a readout that was directly proportional to the number of viable parasites, we compared microscopy counts with ATP levels. This is important to confirm the reliability of the assay. A high degree of correlation was observed (R^2^ = 0.99) as shown in Figure 3.

Then, to validate the assay, 6 common anti-plasmodial drugs such as epoxomicin, dihydroartemisinin, artesunate, primaquine, chloroquine and methylene blue were tested. Dose-response curves and IC_50_ values determined by quantification of the intra cellular ATP were compared to those obtained in parallel using the standard method of microscopic enumeration of gametocytes on stained smears after drug exposure. Cytotoxicity studies were also carried out to investigate the corresponding selectivity ratio (activity in HepG2 cells versus activity against *P. falciparum* gametocytes)

The first compound evaluated, the proteasome inhibitor epoxomicin, is known to have a very potent effect on gametocytes [2]. In the present study, it also exhibited a very high activity in both the ATP bioluminescence assay (0.42 nM) and microscopically (5.2 nM). Nevertheless, IC_50_ values (3 nM) in HepG2 cells were in the same range, suggesting a total lack of selectivity. Inhibition of proteasome activity has a toxic effect on the parasite, but this function is also essential in mammalian cells and, considering the difference of cytotoxicity exhibited by epoxomicin on the HepG2 cell line and on mouse 3T3 or human A549 cells [18], its use may have to be reconsidered.

The two artemisinin derivatives used in our study, dihydroartemisinin (DHA) and artesunate, had no particular activity against mature gametocytes with IC_50_ values of 3.56 µM and 10.83 µM respectively. Activity of artemisinins against these forms is still controversial as very few data have been reported. The effect of artesunate was described in only two studies that found IC_50_ values of 108 nM [19] and 0.1 ng/mL [20]. A single article, describing a new flow cytometry assay [3] , demonstrated an IC_50_ of 1 µM for artemisinin on late gametocytes and its metabolite, DHA, was found to inhibit gametocytes by 70.4% at 1 µM [2]. It is well known that treatment of *Plasmodium* with artemisinin derivatives is associated with lower rates of gametocyte carriage [21], [22] but there is a huge gap in *in vitro* knowledge and additional work is required. Interestingly, the two artemisinin derivatives in the present study showed a high selectivity for the target in asexual stages as IC_50_ values obtained in HepG2 cells exceeded 50 µM.

Primaquine has been reported to selectively destroy the inner structure of *P.falciparum* mitochondria. The mechanism by which primaquine causes its effects on mitochondria remains unclear. It is assumed that primaquine activity depends to a large extent on the formation of metabolites which are more active than the parent compound [23]. This 8-aminoquinoline has long been known to reduce the prevalence of circulating gametocytes in the peripheral bloodstream of parasitemic individuals and to prevent exflagellation among gametocytes present in the test [24], [25]. The IC_50_ on late gametocytes (20.9 µM) and asexual forms (>10 µM) described in this study are in accordance with current literature. Due to the absence of the active metabolites involved in its mechanism of action, primaquine remains inactive *in vitro*. The related 4-aminoquinolines, including chloroquine, affect *P. falciparum* immature gametocytes but not mature gametocytes [26]. Chloroquine kills the *Plasmodium* parasite by inhibiting hemozoin formation and glutathione degradation. Absence of these metabolic reactions at stages IV and V would account for the inactivity of chloroquine reported by Smalley et al. [26] The IC_50_ of chloroquine observed with our ATP bioluminescence assay (23.47 µM) agreed with the values reported in the literature.

Methylene blue (MB) has recently been reconsidered as a useful antimalarial drug [4] and has been explored in combination with CQ and artemisinin derivatives [27]. MB was identified as a specific inhibitor of *P. falciparum* glutathione reductase, and blocks heme polymerization within the food vacuole, is active against all asexual blood stages. Promising data have been reported in clinical trials [28] and, to date, only one publication has shown the *in vitro* activity of MB in mature gametocytes [4]. Our study evaluated for the first time the IC_50_ of MB on stage IV–V gametocytes (0.49 µM) and the IC_50_ on the HepG2 cell line (6.52 µM), and found good activity but quite high cytotoxicity.

**Figure 4:**
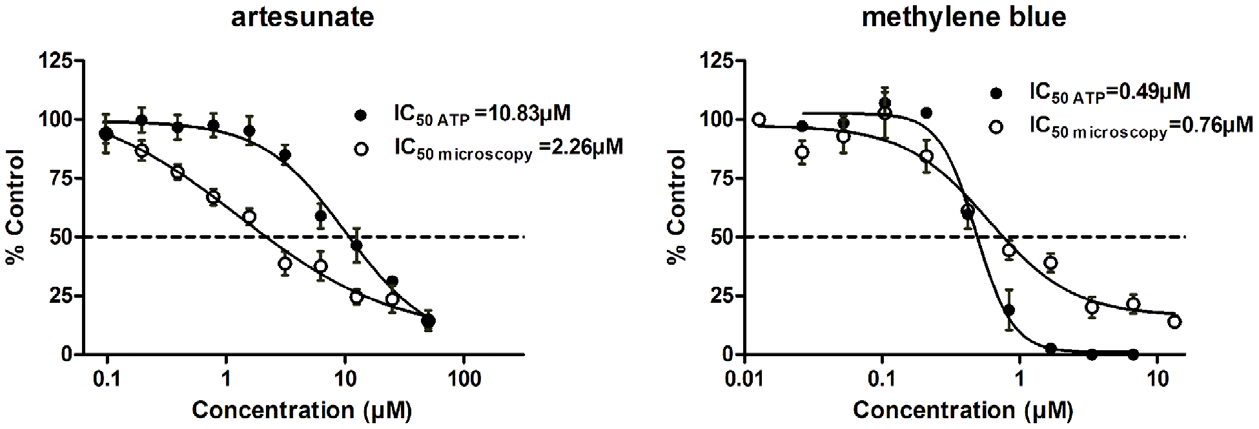
Comparison of the dose-response curves of artesunate and methylene blue obtained with the ATP bioluminescence assay (black dots) or by microscopic counting (open circles). Each point represents mean values of 4 replicates ± SD.

As mentioned earlier, it was essential to determine the activity on mature gametocytes using the gold standard microscopy-based parasite enumeration to validate the assay. As an example, the dose-response curves obtained for both artesunate and methylene blue were represented in Figure 4 and all the IC_50_ results are summarized in Table 1. Microscopy examination showed IC_50_ values comparable to those obtained with the ATP bioluminescence assay in all cases. Differences were expected because one of the drawbacks of the microscopic method is that all parasites are counted, including dead parasites that represent false positives. Even with this shift, the active/inactive profile observed in the dose-response curves calculated from microscopic observations supported the results found with the ATP bioluminescence assay and thus validated this technique.

**Table 1:** Comparison of the activity against mature gametocytes (IC_50_) using either the ATP bioluminescence assay or the microscopical enumeration.

Compounds	IC_50_ gametocytes (µM) Microscopy examination	IC_50_ gametocytes (µM) ATP method
Chloroquine	28.4	23.47
Artesunate	2.26	10.83
Methylene blue	0.76	0.49
Primaquine	87.9	20.9
Dihydroartemisinin	2.47	3.56
Epoxomicin	0.0052	0.00042

### From validation to test

After validation had been performed and based on the differences in mechanisms of action, 10 other drugs currently in use or in clinical development were tested against mature gametocytes using the ATP bioluminescence assay.

The activity against asexual stages was also determined (Table 2) with the reference strain line 3D7A and the 3D7HT-GFP strain to confirm that they both have the same behavior. The activity against mature gametocytes was done using the 3D7HT-GFP strain. Finally, cytotoxicity in the human cell line HepG2 was assessed.

**Table 2:** *In vitro* activity of the compounds against asexual stages, mature gametocytes and assessment of cytotoxicity in the HepG2 cell line.

Compounds	IC_50_ (µM)			
	Activity against asexual forms (3D7A)	Activity against asexual forms (3D7HT-GFP)	Activity against mature gametocytes IV–V	Cytotoxicity
***Quinolines***				
Chloroquine	0.006±0.0002	0.007±0.002	23.47±2.66	51.84±7.86
Primaquine	>10	>10	20.90±4.65	>50
Isoquine	0.009±0.0016	0.0044±0.0032	28.5±2.12	17.07±6.3
Pyronaridine	0.0045±0.0015	0.00080±0.0001	3.25±0.92	5.95±0.92
***Artemisinin Endoperoxides***				
Artesunate	0.037±0.025	0.054±0.03	10.83±3.7	>50
Dihydroartemisinin	0.0028±0.0007	0.013±0.003	3.56±0.95	>50
***Amino alcohols***				
Quinine	0.066±0.018	0.106±0.040	>50	>50
Mefloquine	0.026±0.017	0.028±0.0014	4.70±2.10	11±0.26
Lumefantrine	0.0032±0.00014	0.00165±0.00007	>50	>50
Halofantrine	0.0011±0.0005	0.0012±0.0005	6.70±0.42	>50
***DHFR-TS inhibitors***				
Pyrimethamine	0.0025±0.0009	33.66±6.72*	>50	>50
***Hydroxy-naphthoquinones***				
Atovaquone	0.0007±0.0004	0.0018±0.0002	16.10±2.12	>40^a^
***Proteasome inhibitors***				
Epoxomicin	0.008±0.001	0.0098±0.0017	0.00042±0.00011	0.003±0.0005
***Dyes***				
Methylene blue	0.011±0.008	0.010±0.0018	0.49±0.16	6.52±1.31
***Protein synthesis inhbitors***				
Azythromycin	>10	>10	28.45±0.64	>50
Cycloheximide	0.198±0.0017	0.194±0.0077	6.23±2.46	2.95±0.35

Inhibition of uptake of a radiolabeled nucleic acid precursor by the parasites served as the indicator of antimalarial activity against asexual stages while the activity on mature gametocytes was determined with the ATP bioluminescence assay. Finally, cytotoxicity was assessed using the short-term resazurin-based reductase assay. All the results are expressed as the average value of the IC_50_ ± standard deviation (3–6 independent experiments).

a limit of solubility.

* resistance to pyrimethamine due to a selection cassette in the episome (containing the GFP gene).

Among the 10 newly tested compounds, quinine and mefloquine, were first investigated. As with other quinoline anti-malarial drugs, the mechanism of action of quinine has not been fully elucidated. The most widely accepted hypothesis about quinine action is based on data for the closely related drug, mefloquine [29], [30]. These data suggest that its main mode of action may be to inhibit hemoglobin ingestion by blocking the endocytotic process [31]. Although they are active against asexual blood stages of chloroquine-resistant (CQR) and sensitive (CQS) *P. falciparum* strains [32], [33], [34], neither quinine (IC_50_ above 50 µM) nor mefloquine (IC_50_ = 4.7 µM) showed good activity on late gametocytes. Moreover, the selectivity ratio of mefloquine (2.34) was quite narrow (IC_50_ of 11 µM in HepG2 cells versus IC_50_ of 4.9 µM in gametocytes)

Several of the compounds that act on hemoglobin metabolism were also evaluated; halofantrine, lumefantrine and isoquine. The exact mechanism by which halofantrine and lumefantrine exert their anti-malarial effect is not well defined. However, available data suggests that halofantrine binds to plasmepsin, a hemoglobin-degrading enzyme and to haematin *in vitro* and lumefantrine inhibits the formation of ß-haematin by forming a complex with haemin. Isoquine, on the other hand, was developed as a potentially safer alternative to amodiaquine, an effective but toxic substitute of chloroquine. Halofantrine, isoquine and lumefantrine (used in the “co-artemether” combination) are active on asexual blood stages of CQR strains [32], [35], [36] but no significant activity on mature gametocytes was detected (IC_50_ = 6.7; 28.5 and >50 µM, respectively) [37]. Lumefantrine and halofantrine were less cytotoxic than isoquine *in vitro* with an IC_50_ higher than 50 µM for both, compared to 17.07 µM for isoquine.

Pyronaridine-artesunate is a promising new artemisinin-based combination therapy for the treatment of uncomplicated *falciparum* malaria. Pyronaridine is also thought to inhibit parasite development because of its ability to inhibit β-haematin formation. It has been shown to have a high *in vitro* activity against chloroquine-sensitive and chloroquine-resistant strains of *P. falciparum* [38]. Moreover, some activity against gametocytes has been reported [39] but what was probably seen was an effect on young gametocytes because the IC_50_ for mature gametocytes was approximately 3.25 µM with the ATP bioluminescence assay.

The antifolate family is an important group of antimalarials of which pyrimethamine is a crucial member. The compound was introduced here as an internal control as the strain *P. falciparum* 3D7HT-GFP contains a selection cassette giving resistance to drugs acting on DHFR. The drug, as expected, did not have any effect on mature gametocytes of this resistant line.

It should be noted that atovaquone, which is often used today in the atovaquone-proguanil combination (Malarone), showed very strong *in vitro* activity against asexual erythrocytic *P. falciparum* cultures (IC_50_ = 1.8 nM) but had no activity against gametocytes according to our ATP assay (IC_50_ = 16.10 µM) and previous reports [19]. It has been shown that atovaquone does inhibit transmission [37], [40] but has no effect on mature gametocytes [41]. No cytotoxic effects were detected at the maximum solubility of atovaquone in culture media.

Use of antibiotics was also investigated. Cycloheximide is an inhibitor of protein biosynthesis in eukaryotic organisms that interferes with the translocation step in protein synthesis. Experimental evidence of antimalarial activity is available for this drug [42] but it showed low activity when tested on stage IV–V gametocytes (IC_50_ = 6.23 µM), as described previously [43]. Asexual forms of the parasite may also be killed by targeting the apicoplast. Azithromycin is a macrolide antibiotic derived from erythromycin. It is not yet licensed for use as an antimalarial agent but has shown promising activity against *P. falciparum in vitro* [44]. This drug appeared to have some effect upon parasite development in the mosquito stage [45] but had no effect upon mature gametocytes, in the present study (IC_50_ = 28.45 µM) and in other experiments [46].

## Discussion

The eradication of malaria will remain an elusive goal unless drugs are found that either massively reduce or, even better, fully inhibit disease transmission. The lack of tools to assess the activity of compounds against the key actors, mature gametocytes, is the real problem at present. Some attempts have been made [2], [3], [4], [14], but published methods are still labor-intensive, costly and/or complex, making development of such a large-scale screening difficult.

Compared to previous methods that assessed the activity of new chemical entities against mature gametocytes, the technique that we have devised has several advantages. This ATP bioluminescence assay is highly sensitive; the luminometric technique is able to detect a low number of cells (1×10^4^ per well in a 384-well plate) even if these cells display a low metabolic activity. This method measures the whole well in the microtiter plate, representative of a population of parasites. When samples are analyzed by microscopy, only an aliquot is taken from the well to prepare a smear and only some fields are counted at random. In addition, our methodology does not rely on operator expertise and is therefore not subject to person-to-person variability. Uncertainty about the viability of parasites when counting is overcome by this new method as only metabolically active parasites are detected. Reading of luminescence is fast, requiring a minimum number of handling steps and providing results within 15 minutes. The plate is read instantaneously and there is no need for time-consuming incubation, as required by other techniques (e.g. alamar blue). An important advantage is that the method can be used to directly evaluate the activity on any parasite strain or clinical isolates as it does not require the use of any parasite line transformed with reporter genes. Moreover, this system is amenable to adaptation to 384-well plates allowing the testing of a higher number of compounds than with traditional manual counting or the flow cytometry technique.

In summary, the work described here provides, for the first time, data on the activity of all clinically relevant antimalarial drugs against mature gametocytes and represents a significant step towards development of a high-content method suitable for high-throughput screening of new molecules at these stages. Considering that the biology of the forms involved in the sexual and asexual stages is very different, a large-scale high-throughput screen using the GlaxoSmithKline 2 million-compound library may allow us to discover innovative anti-malarial drugs to target novel transmission-specific metabolic pathways.

## Methods

### Parasites and Cultures

Three strains of *Plasmodium falciparum*, W2-Indochina (MR4, MRA-157), 3D7A (MR4, MRA-151) and the 3D7HT-GFP strain [15], kindly provided by Robert E. Sinden, were cultured using a method modified from that of Trager and Jensen [47] in a 5% CO_2_/5% O_2_ atmosphere at 37°C [48].

### Gametocytogenesis

Gametocyte cultures of each strain were initiated as described by Ifediba and Vanderberg [8], but with the following minor variations: On day 0, cultures were synchronized at the ring stage by lysis of the flask pellet with 5 volumes of 5% sorbitol for 10 min at 37°C. Cultures were then initiated at 0.2% parasitaemia and 12% hematocrit in a 10 mL volume. The culture was first incubated in RPMI 1640 (Gibco) supplemented with hypoxanthine (Sigma-Aldrich), sodium bicarbonate (Sigma-Aldrich) and 15% AlbuMAX II solution. On day 8, the volume was doubled and the concentration of AlbuMAX was reduced to 10%. Finally, on day 12, cultures were treated with 50 mM *N*-acetyl-D-glucosamine (GLcNA, Sigma-Aldrich) and 50 ng/mL of bistratene A for 3–5 days to remove asexual forms. Medium was changed daily throughout the process. To assess gametocytogenesis curves (Fig. 1), the percentage of asexual forms and gametocytes were counted on Giemsa-stained smears.

### Purification

On day 15 (Fig. 1), the majority of the gametocytes were stage IV–V. At this point, parasites were concentrated with NycoPrep™ 1.077 cushions (Axis-Shield). The culture pellet was washed, resuspended in 10 mL of medium and placed slowly onto 5 mL of Nycoprep cushion in a 15 mL Falcon tube. The tube was centrifuged at 800 g for 20 minutes. The band was collected, washed, and spun down and the pellet was resuspended in 5 mL of medium. During the entire procedure the gametocytes were maintained at 37°C. A Giemsa-stained smear was prepared at this point. The solution containing the gametocytes was then loaded onto a LS-Column (Miltenyi Biotech) to carry out the second purification step using a VarioMACS magnetic separator (Miltenyi Biotech). The column was washed with 5 mL of medium to elute the remaining RBCs and, finally, gametocytes were immediately flushed out by removing the column from the holder, adding 5 mL of medium, and firmly applying the plunger supplied with the column. On average, 2.5 to 10 million gametocytes were obtained from a 25 cm^2^ culture flask.

### IC_50_ assays

Compounds used for the validation included dihydroartemisinin (Sigma-Aldrich), artesunate (Apin Chemicals Limited), epoxomicin (Sigma-Aldrich), primaquine (Sigma-Aldrich), methylene blue (Sigma-Aldrich) and chloroquine (Sigma-Aldrich). The first four compounds were dissolved in DMSO, while chloroquine and methylene blue were dissolved in water.

#### ATP bioluminescence assay

Once purified, gametocytes were counted using a Neubauer chamber. Gametocytes (5×10^4^ gametocytes per well) were transferred to a 96-well plate in a final volume of 100 µL, and dilutions of each drug were added (final DMSO concentration 0.5%). Plates were incubated (at 37°C in a 5% CO_2_ humidified incubator) for 48 h. The ATP level of each well was determined using the BacTiter-Glo™ reagent (Promega) according to manufacturer's recommendations. The BacTiter-Glo™ assay generates a “glow-type” luminescent signal produced by the luciferase reaction, which consists of mono-oxygenation of luciferin catalyzed by luciferase in the presence of Mg2+, ATP, and molecular oxygen.

#### Microscopic measurement of IC50 values

IC_50_ values were determined after 48 h of incubation with different dilutions of the drugs. 100 µl of the culture (day 15, HT 6%) were plated in each well with the appropriate dilution of drug. 48 h after, 10 µl of each well was used to prepare a thin blood film and stain it with Giemsa. The number of gametocytes per 10,000–15,000 RBCs were counted and compared to the control (no drug, 0.5% DMSO).

#### In vitro antiplasmodial activity on asexual stages

IC_50_ values were measured as previously reported [49] but all of the tests were done with serum-free cultures (using AlbuMAX II)

### Cytotoxicity

#### Cell line

HepG2, a human caucasian hepatocellular carcinoma, was supplied by ECACC (ref. 85011430).

#### Routine culture

Cells were grown and maintained in EMEM (Sigma-Aldrich) supplemented with 2 mM L-glutamine (Sigma-Aldrich) and 10% fetal calf serum (Perbio). Cultures were maintained at 37°C in a humidified incubator containing 5% CO_2_, 95% air and passages were routinely made upon reaching 80% to 90% confluence. For cytotoxicity experiments, cells were seeded onto 96-well clear bottom black plates coated with type I collagen (Biocoat, Becton Dickinson) at a cell density of 10000 cells/well.

#### Measurement of cytotoxicity

To determine cytotoxic effects, represented by the IC_50_ value (the concentration of drug that reduces cell viability by 50%), cells were exposed to serial dilutions of test compounds for 48 h at 37°C. The culture medium was as described above, but supplemented with 5% fetal calf serum. Following the 48 h exposure period, a 0.004% resazurin solution was prepared by adding 60 mL of Dulbecco's PBS to each resazurin tablet (VWR International). The tablet was allowed to dissolve by placing the container in a bath maintained at 37°C, protected from light, for approximately 30 minutes. Medium was removed and 200 µL of fresh culture medium and 50 µL of resazurin solution were added to each well. Plates were incubated for a further 1 ½ hours. Cytotoxicity is indicated by decreased reduction of resazurin to its fluorescent product resorufin. The fluorescence was stabilized at room temperature for 15 minutes protected from light. Fluorescence was measured using a fluorescence plate reader (Victor V, Perkin Elmer) at an excitation wavelength of 515 nm and an emission wavelength of 590 nm. Percentages of inhibition were calculated relative to the control wells.

#### Selectivity ratio

We defined the selectivity ratio as the IC_50_ value in HepG2 cells/IC_50_ value in *P.falciparum*. For a late lead or a preclinical candidate, a differential sensitivity between host and parasite at cellular level higher than 100-fold is considered acceptable.
